# Relationship between midwifery educators’ quality of life and students’ clinical training quality

**DOI:** 10.1186/s12909-026-09637-2

**Published:** 2026-06-04

**Authors:** Ebrahim Abbasi, Mahboubeh Abbasifar, Lila Bazrafkan

**Affiliations:** 1 Medical Education Department, Medical Education Development Center, Shiraz University of Medical Sciences, Shiraz, Iran; 2 Department of Biology and Control of Disease Vectors, School of Health, Shiraz University of Medical Sciences, Shiraz, Iran

**Keywords:** Adult Learning, Collaborative Learning, Evaluation Methodologies, Pedagogical Issues

## Abstract

**Introduction:**

Clinical education is a fundamental component of midwifery training that enables students to apply theoretical knowledge in real clinical environments. However, several challenges such as the dispersion of internships across departments, large student-to-instructor ratios, limited instructor accessibility, poor coordination between theoretical and clinical education, and stressful clinical conditions can reduce its effectiveness. Since educators’ personal well-being may influence their teaching performance, this study aimed to examine the relationship between the quality of life of midwifery educators and the quality of clinical education of midwifery students in Fars Province, Iran.

**Materials and methods:**

Before conducting this cross-sectional descriptive–analytical study, a brief search of domestic and international databases (SID, Magiran, Google Scholar, Scopus, PubMed, and Web of Science) was performed using the keywords “quality of life of educators”, “quality of clinical education”, and “midwifery”, which revealed no previous research directly addressing this relationship. The study was therefore designed to fill this gap. Participants included 94 midwifery educators and 181 final-year midwifery students from seven universities in Fars Province. Data were collected using three instruments: a demographic questionnaire, the SF-36 Quality of Life Questionnaire (completed by educators), and the Clinical Education Status Questionnaire (completed by students). Data were analyzed with SPSS version 24 using descriptive statistics and Spearman’s correlation test due to non-normal data distribution.

**Results:**

The mean total quality-of-life score of educators was 70.03 ± 13.4, and the mean clinical education quality score of students was 96.98 ± 10.2. A significant positive correlation was found between educators’ quality of life and students’ clinical education quality at the institutional level (*r* = 0.219, *p* = 0.042), indicating an association between educator well-being and students’ perceptions of clinical education within the same universities, indicating that better educator well-being was associated with higher perceived educational quality. Additionally, students’ age was inversely correlated with their perception of clinical education quality (*r* = − 0.259, *p* = 0.001). Among the eight dimensions of quality of life, physical performance was the strongest predictor of clinical education quality (β = 0.807, *p* < 0.01).

**Conclusion:**

The findings indicate that midwifery educators’ quality of life is positively associated with the quality of clinical education perceived by students within the same institutional settings. Enhancing the physical and psychological well-being of educators may therefore improve the overall effectiveness of midwifery training programs and lead to higher-quality clinical education.

## Highlights

The study identifies multiple challenges in clinical education for midwifery, such as the dispersion of internships, overburdening students with employee duties, large student numbers, inadequate instructor evaluations, limited access to trainers, poor coordination between theoretical and clinical training, insufficient time for practicing learned skills, and stressful situations.

A comprehensive review of existing literature was conducted through searches in domestic and international databases like SID, Magiran, Google Scholar, Scopus, PubMed, and Web of Sciences using keywords such as "quality of life of educators," "quality of clinical education," and "midwifery." No direct studies on the correlation between midwifery educators' quality of life and clinical training quality were found. Hence, related studies in other disciplines were reviewed.

Spearman's non-parametric correlation test was employed to investigate the hypothesis that there is a significant correlation between the quality of life of educational instructors and the clinical training quality of midwifery students. Another correlation test examined the relationship considering the age variable of respondents.

The study's findings reveal an inverse and significant correlation between the clinical education quality of midwifery students and their age. Overall, a positive and significant correlation exists between the clinical training quality of midwifery students and the quality of life of their educational instructors, as indicated by the correlation coefficient.

## Introduction

Midwifery is one of the medical professions and one of the vital fields in the health care system. The education of this field is a collection of theoretical sciences and practical and skillful activities. In practice, more than 50% of midwifery students’ time is spent in bedside clinical training, and bedside education is therefore a vital component of their professional preparation [1]. Medical science education is the infrastructure for providing efficient human resources in the field of health; It includes two main parts theoretical and clinical education. Clinical training is the connecting link between the theoretical content of medical students and providing clinical services to patients. Clinical education can be considered as an activity that facilitates learning in the clinical environment, in which the clinical instructor and the student participate equally, and its purpose is to create measurable changes in the student to perform clinical care [2, 3].

Clinical education is a structured process in which students acquire practical skills by working directly with patients under supervision. It bridges theoretical knowledge with clinical application and aims to develop confidence, critical judgment, and problem-solving abilities. Through guided practice, students prepare to provide independent, competent patient care, which represents a measurable outcome of successful clinical instruction. It is to recruit students to perform clinical care [4, 5]. In general, the purpose of clinical activities and experiences in clinical education is to provide confidence and independence in performing therapeutic measures, critical judgment, and problem-solving, which is possible through the application of theoretical knowledge in practice. Normally, medical schools combine theoretical training programs with clinical or practical training so that the education of students is complete [6]. Considering the rapid changes in healthcare environments, the more productive the clinical training is, the more students’ efficiency will be.

Today, the policymakers of the World Health Organization acknowledge the importance of the midwifery profession and it’s training more than ever before [7]. The aim of midwifery education is to train committed experts in the field of midwifery education and services. So that they can provide and maintain the health of mothers and children in society with the necessary efficiency and skill and provide extensive quality health and midwifery services. Therefore, improving the quality of midwifery education is vital for improving the health of mothers and babies [8]. The quality of education is one of the concerns that university systems always strive to achieve. In recent decades, significant efforts have been made in the field of continuous improvement of the quality of higher education and achieving the goals of university systems. The quality of education is one of the most important factors in improving the scientific level of societies and the ranking of universities; along with the two factors of cost and time, it is considered the three basic factors of universities and higher education institutions, but the quality has received serious attention more than the other two factors, and if the quality is improved, the costs decrease and productivity increases [9].

During the studies conducted, clinical education in midwifery has a series of problems, which are generally factors such as the dispersion of internships in clinical departments, forcing students to perform the duties of employees, large number of students, lack of proper evaluation by the instructor, lack of Sufficient access to trainers, lack of proper coordination between theoretical and clinical training, lack of time needed to face different cases of disease to fully practice what was learned in the clinical environment and stressful situations can be mentioned among the problems of clinical training [10]. Among the most important components of clinical education, we can point out such things as comprehensive individual characteristics, instructor, training environment, planning, and evaluation [4]. Meanwhile, the characteristics of trainers during clinical training are considered an important feature in the quality of training. During a study, it was shown that the characteristics of a good teacher, in order of importance, include knowledge, personality, communication, teaching method, evaluation, and ethics, which were the most important factors [11]. Although the terms quality of life and quality of work life are sometimes used interchangeably, they represent different concepts. Quality of life (QOL) encompasses a person’s overall physical health, psychological state, social relationships, and perception of well-being within the context of their culture and environment. In contrast, quality of work life (QWL) focuses specifically on satisfaction and well-being within the workplace. Because this study utilized the SF-36 Health-Related Quality of Life Questionnaire which measures general well-being rather than job-specific satisfaction the intended concept in this research is quality of life. Understanding educators’ overall quality of life is essential, as it influences their energy, motivation, communication, and emotional stability, all of which can directly affect the teaching–learning process in clinical education [12].

Quality of life is a broad concept and is complexly related to physical health, psychological status, degree of independence, social relationships, personal beliefs of a person, and environmental factors. Quality of life is a multidimensional concept, relative and affected by time and place and individual and social values [13]. Humans increasingly tend to increase their lifespan and pay special attention to the positive features of life such as quality of life and consider quality of life in their lifestyle. These factors have an impact on life and play a role in the formation of interpersonal relationships [14]. The World Health Organization defines quality of life as the understanding of each person about his life situation according to the culture and value systems in which he lives and its relationship with the goals, expectations, standards, and priorities of the individual [15]. Examining the quality of life or the degree of people’s feelings about their abilities regarding physical, emotional, and social functions has been raised as an important issue in health care for more than a decade [16]. The relationship between the quality of life of educators and the quality of midwifery education lies in the fact that instructors with higher well-being are better able to engage students, manage clinical environments, and model professional behavior. Fatigue, stress, or low psychological well-being can impair teaching effectiveness and reduce students’ learning experiences. Therefore, exploring this relationship provides valuable insights into how supporting educators’ health and life satisfaction can enhance the quality of clinical education for midwifery students.

The quality of life of professors serves as the foundation for better and higher-quality education in universities, and this will lead to the cultivation of successful students and the implementation of practical and effective projects [17].The quality of life causes the alignment of employees and the organization; besides, many job attitudes such as job satisfaction, organizational commitment, and job attachment affect people’s personal, family, and social lives [18]. Comparing the quality of life of educators with the quality of clinical education of students is essential because the well-being of instructors can directly influence their professional performance and interactions with learners. Educators with higher quality of life tend to experience better mental and physical health, leading to more effective communication, higher teaching motivation, and more positive educational environments. Consequently, the quality of educators’ lives may be reflected in the educational experience of their students, especially in clinical training where close instructor-student interactions are vital. This comparison provides insight into how personal well-being can translate into professional teaching quality. Therefore, the quality of life is considered an important variable among trainers and professors. Considering the multifactorial nature of clinical education and the potential role of educators’ personal well-being as one contributing element among many, this study was conducted to explore the association between the quality of life of midwifery educators and the perceived quality of clinical education among midwifery students in Iran.

## Materials and methods

### Review of previous studies

To review existing literature related to this topic, a comprehensive search was conducted in both domestic and international databases, including SID, Magiran, Google Scholar, Scopus, PubMed, and the Web of Science. The search covered publications from 2000 to 2023 using the following English and Persian keywords in various combinations: “quality of life of educators”, “quality of work life”, “quality of clinical education”, “midwifery education”, “nursing education”, and “clinical training quality”. The inclusion criteria consisted of (1) studies involving educators or faculty members in the fields of midwifery, nursing, or medical sciences; (2) studies evaluating either the quality of life or the quality of clinical education; and (3) articles published in peer-reviewed journals. Exclusion criteria were (1) studies not available in full text and (2) studies unrelated to educational or health science contexts. The search yielded several studies examining the quality of clinical education from students’ perspectives (e.g., Delaram, 2006 [19]; Rezaei, 2016 [4]; Ghafourifard, 2016 [20]) and the quality of life or work life of educators and university faculty members (e.g., Rezaei et al., 2020 [21]; Yang et al., 2009 [22]; Janalizadeh & Farzaneh, 2014 [23]; Akar, 2018 [17]). However, no previous studies were found that specifically addressed the relationship between midwifery educators’ quality of life and students’ clinical education quality. Therefore, the current research was designed to fill this identified gap by empirically investigating the connection between these two variables in Fars Province [24, 25].

### Type of study

This study is a cross-sectional descriptive-analytical study, which was conducted in 1401 on midwifery instructors and students in the universities of Shiraz, Lar, Firozabad, Esteban, Arsenjan, Kazeroon, and Jahrom, with the aim of investigating the relationship between the quality of life of the instructors. Midwifery and the quality of clinical training of midwifery students was done.

### Research environment

The research environment in this study is the universities of Shiraz, Lar, Firouzabad, Estehban, Arsanjan, Kazeroon and Jahrom.

### Research community

The research community of this study included all midwifery trainers and midwifery students of Fars province. Therefore, this study had two statistical populations; In this way, the quality-of-life variable in midwifery instructors in the universities of Shiraz (15 people), Lar (14 people), Firozabad (15 people), Estehban (10 people), Arsanjan (15 people), Kazerun (15 people) and Jahrom (10 people) was measured, the statistical population was equal to 94 people. Also, the quality of clinical education from the point of view of final-year midwifery students in the universities of Shiraz (18 people), Lar (23 people), Firozabad (35 people), Estehban (25 people), Arsanjan (25 people), Kazerun (30 people) and Jahrom (25 people) whose statistical population was equal to 181 people. The statistical population of midwifery professors and students in different universities and cities was obtained by correspondence with the head of the department of each city and can be cited.

### Examples of research

This study was conducted as a census; Therefore, the size of the research samples was equal to the entire statistical population, 94 midwife educators and 181 midwife students.

### Characteristics of research units

Inclusion Criteria.

Eligibility criteria for educators.

- Trainers in midwifery.
- Instructors who directly give clinical training to students.
- Have at least one year of clinical teaching experience.

### Inclusion criteria in students

In Iran, midwifery education is offered at both undergraduate (bachelor’s) and postgraduate (master’s) levels. The undergraduate program lasts four years and includes extensive clinical and theoretical training, with the final year emphasizing practical internships.

- Final year midwifery students.

### Exclusion criteria

- Participants’ unwillingness to cooperate in completing the questionnaires.

### Information gathering tool

In order to collect information, a demographic researcher questionnaire, SF-36 health-related quality of life questionnaire, and clinical education status questionnaire from the student’s point of view were used.

1. Demographic Information Questionnaire: This researcher-made questionnaire was developed to collect basic personal and professional characteristics of participants. It included six items for educators (city of service, age, level of education, marital status, work experience, and type of employment) and four items for students (city of education, age, level of education, and marital status). To ensure content validity, the initial draft of the questionnaire was reviewed by five experts in medical education and midwifery from Shiraz University of Medical Sciences. They assessed each item for clarity, relevance, and comprehensiveness. Based on their feedback, minor revisions were made to improve the wording and content of the questions. The final version was approved by the expert panel. In this questionnaire, the term education refers specifically to the highest academic degree obtained for educators (e.g., Bachelor’s, Master’s, or Ph.D.) and the current educational level or program for students (e.g., undergraduate or master’s level).
2. Quality of life questionnaire: SF-36 health-related quality of life questionnaire is a standard questionnaire that was completed by the trainers. This questionnaire was created by Weber in 1992, and it is for the purpose of evaluating health policies and generally evaluating the state of health in terms of physical and mental status; it has 36 questions and 8 components, the components of which include physical performance, role performance limitations due to physical health status, role performance limitations due to emotional problems, energy and vitality, emotional health, social performance, pain and it is public health. Based on the Likert scale, this questionnaire measures the health-related quality of life with questions such as (compared to last year, how do you evaluate your current health in general). Also, the second part of the five-point Likert scale includes (completely false, somewhat false, I don't know, somewhat true, completely true). The validity and reliability of this questionnaire in Montazeri's research and collaboration and Cronbach's alpha coefficient for this questionnaire have been estimated above 0.7 [26].

Questionnaire on the status of clinical education from the student’s point of view: This questionnaire is a standard questionnaire that was completed by the students. This questionnaire has 33 questions and its purpose is to evaluate the state of clinical education from the student’s point of view (educational objectives and program, instructor, dealing with students, educational environment, monitoring, and evaluation). The scoring of the questionnaire is on a 5-point Likert scale: very low, low, medium, high, and very high. In the research of Del Aram et al. (2006), the validity of this scale has been confirmed by the relevant professors. Also, the reliability of this questionnaire has been confirmed using Cronbach’s alpha (above 0.70 for the dimensions of the questionnaire) [26].

### Research implementation method

After obtaining the necessary permits and obtaining the approval of the ethics committee from the research vice-chancellor of the university, it was implemented by referring to various faculties and introducing letters to the officials of the department. The desired sample will be selected based on the accessible method. After identifying the desired people, including students and teachers, they were invited to participate in the study; And informed consent was obtained from them to participate in the study. Then the desired questionnaires were given to midwifery students and trainers and this questionnaire was completed by the samples. After collecting the data, it was analyzed by SPSS version 24 software and statistical tests [27, 28].

### Data analysis

After collecting primary information by questionnaire, these data were coded and analyzed using SPSS version 24 statistical software. Descriptive statistical methods such as average indices, standard deviation, frequency, percentage, etc., were used based on the type of variable to describe the investigated variables. Mann-Whitney statistical tests and Spearman’s correlation coefficient were also used to compare the data. Because educators and students were recruited from multiple universities across different cities, direct individual-level matching between specific instructors and specific students was not feasible. Instead, analyses were conducted at the institutional (city/university) level. Educators’ quality-of-life scores were aggregated within each university, and students’ clinical education quality scores were likewise aggregated within the same institutions. Correlation analyses were then performed using these institution-level aggregated scores to examine the association between educators’ quality of life and students’ perceived quality of clinical education within the same educational settings. All educators included in the study were confirmed to be actively involved in clinical teaching of the participating final-year students within their respective universities.

## Results

### Descriptive findings

#### Gender of students

Based on the obtained results, 183 midwifery students of Shiraz participated in this research in undergraduate and graduate courses, all of them were women, so gender is not considered as a variable in this research.

### Age variable

Based on the obtained results, 87 educational instructors and 183 students participated in this research in undergraduate and postgraduate courses in midwifery at Shiraz University of Medical Sciences. The descriptive statistics of the age variable of the respondents are also shown in Table 1.

**Table 1:** Frequency distribution

Frequency distribution of the research respondents according to age					
Age	Number	At Least	Maximum	Average	Standard Deviation
Students	183	18	38	25.01	4.87
Teachers	87	22	54	40.85	6.4

Frequency distribution of midwifery students according to educational level					
Grade		Abundance	Percent		
Bachelor Degree		140	76.5		
Master Degree		43	23.5		
Total		183	100		

Regarding the distribution of the respondents according to the age variable, the research findings indicated that the youngest and oldest students were 18 and 38 years old, respectively, and their average age was 25.01. The youngest and oldest trainers were 22 and 54 years old, respectively, and their average age was 40.85 (Table 1).

### Grade

Regarding the distribution of the number of students by educational level, the findings of the research indicated that 140 students, equivalent to 76.5% of the total respondents, were undergraduate students and 43 master’s students, equivalent to 23.5% of the sample size of this research. Give (Table 1).

### Describing the quality of clinical education from the students’ point of view and its dimensions

The quality of clinical education from the students’ perspective was assessed using the standardized questionnaire developed by Delaram et al. (2006), which evaluates students’ perceptions of educational objectives, instructor performance, student–instructor interactions, educational environment, and monitoring and evaluation. This questionnaire has 33 questions and its purpose is to evaluate the state of clinical education from the student’s point of view (educational objectives and program, instructor, dealing with students, educational environment, monitoring, and evaluation). The scoring of the questionnaire is on a 5-point Likert scale: very low, low, medium, high, and very high (Table 2). The students’ total evaluation score was 98.96 out of 165 points, which is above the average level. The results indicated that the students assigned the highest scores to the dimensions of instructor performance and monitoring and evaluation, respectively. In contrast, the *educational environment* dimension received the lowest score in this assessment.

**Table 2:** Description of midwifery students’ assessment of the quality of clinical education and its dimensions

Age variable	At least	maximum	average	Percent	Total percentage
Quality of education	52	165	96.98	56.78	56.78
Goals and plans	11	55	31.19	56.71	19.33
Coach performance	14	45	28.77	63.93	21.80
Dealing with students	3	15	8.57	57.13	19.48
Learning environment	7	35	19.44	55.54	18.94
Monitoring and evaluation	3	15	9	60	20.46
Quality of Life			70.03	70.03	70.03
Physical Performance			23.46	78.20	13.99
Limitations due to physical health			5.77	72.12	12.90
Limitations due to emotional problems			4.37	72.83	13.03
Energy and vitality			8.75	62.50	11.18
Emotional Health			13.17	65.85	11.78
Social Performance			4.11	68.50	12.25
The Pain			1.51	75.50	13.51
General Health			8.89	63.50	11.36

#### Description of the quality of life of educational coaches and its dimensions

The quality of life of educational trainers was measured using the SF-36 Health-Related Quality of Life Questionnaire (Ware, 1992). This standard tool contains 36 items and evaluates eight dimensions of health-related quality of life: physical functioning, role limitations due to physical health, role limitations due to emotional problems, vitality, emotional well-being, social functioning, bodily pain, and general health. Each item is scored on a Likert scale, and the total score is converted to a 0–100 scale, where higher scores indicate better quality of life. The validity and reliability of the Persian version of this instrument were confirmed in the study by Montazeri et al., with Cronbach’s alpha coefficients exceeding 0.70 (Table 2).

### Analytical finding

#### Check the status of the new data

The quality of life (health quality variable) of educational instructors was assessed using the SF-36 Health-Related Quality of Life Questionnaire (Ware, 1992). This standardized instrument consists of 36 items across eight domains: physical functioning, role limitations due to physical health, role limitations due to emotional problems, vitality, emotional well-being, social functioning, bodily pain, and general health perceptions. Each domain score ranges from 0 to 100, with higher scores indicating better quality of life. The reliability and validity of the Persian version of this questionnaire have been confirmed in previous studies, with Cronbach’s alpha values above 0.70. Kolmogorov-Smirnov test was used to check the distribution of data. The results of this test showed that the data distribution in the health quality variable of educational instructors does not follow the normal distribution and the data distribution is non-normal, therefore, non-parametric tests were used to check the hypotheses of this research.

- Hypothesis: There is a significant correlation between the level of quality of life of educational instructors and the level of clinical training of midwifery students.

Investigate the research hypothesis that “there is a significant correlation between the level of quality of life of educational instructors and the level of clinical training of midwifery students.” Spearman’s non-parametric correlation test was used, the results of this test are presented in Table 3.

**Table 3:** Examining the distribution of data related to research variables

Kolmogorov-Smirnov test	Quality of student education	The quality of coaches’ life	
Test amount	0.043	0.128	
Significant level	0.2	0.001	
Number	183	87	
Correlation test to determine the relationship between the quality of life of educational instructors and the level of quality of education of students.			
Variables	Number	Spearman’s correlation coefficient	Meaningful
Clinical training of students	183	0.219	0.042
The quality of coaches’ life	87		

The Table 3 shows that according to the correlation coefficient, there is a positive and significant correlation between the variable of midwifery students’ clinical training and the level of quality of life of their educational instructors (*r* = 0.219, *p* = 0.042). In other words, in parallel with the increase in the quality of life of educational instructors, the level of clinical education of students has also increased and vice versa. Assumption: There is a significant relationship between the quality of life of educational coaches and their work experience.

In order to investigate the research hypothesis that “there is a significant correlation between the quality of life of educational coaches and their work history.” Spearman’s non-parametric correlation test was used, the results of this test are presented in Table 4. Based on the coefficients of the correlation test, there is no significant correlation between the quality of life of trainers and their work experience. (*r* = 0.179, *p* = 0.098)

##### Assumption

There is a significant correlation between the quality of life of educational instructors and the educational quality of students with the age variable of the respondents. In order to investigate the research hypothesis that “there is a significant correlation between the quality of life of educational instructors and the educational quality of students with the age variable of the respondents.” A correlation test was used, the results of this test are presented in Table 4.

**Table 4:** Correlation test

Correlation test to determine the relationship between the quality of life of educational coaches and their work experience		
Variables	Spearman’s correlation coefficient	Meaningful
The quality of coaches’ life	-0.179	0.098
Work history of coaches		

Correlation test of teachers’ quality of life and students’ educational quality with the age variable of the respondents.		
Correlation test	Clinical training of students	The health of the coaches
Test amount	-0.259	-0.128
Significant level	0.001	0.238
Number	183	87

The Table 4 shows that according to the correlation coefficient (*r* = 0.259, *p* = 0.001), there is an inverse and significant correlation between the variable of clinical education quality of midwifery students and their age level. In other words, as the educational quality level of midwifery students increases, the age level of students decreases and vice versa. Therefore, younger students have a higher quality of clinical education than their older peers.

According to the results of the correlation test, there is no significant correlation between the quality of life of coaches and their age level. (*r* = 0.128, *p* = 0.238). Assumption: the components of the quality of life of educators are considered to be suitable predictors for the criterion variable of the quality of students’ education. Spearman’s correlation was used to check this assumption. The results are presented in the form of Table (8), and by referring to the results of the table, it can be acknowledged that there is a significant correlation between the quality of life of educators and its components with the quality of students’ education (Table 5).

**Table 5:** Spearman’s correlation coefficient between the quality of life of instructors and the quality of students’ education

Variable	Quality of education	The significance level
Physical Performance	0.441^b^	0.000
Emotional Health	0.028	0.799
The Pain	^a^0.267	0.013
General Health	-0.43	0.690
Energy and Vitality	0.013-	0.901
Social Performance	0.083	0.443
Limitation due to emotional problems	0.310^b^	0.003
Limitation due to physical health	0.317^b^	0.003

^a^ Indicates that the correlation coefficient is significant at the 0.05 level. ^b^ indicates that the correlation coefficient is significant at the 0.01 level

In order to investigate the predictive power of the quality-of-life components of educators (physical performance, emotional health, general health, etc.) in the quality of students’ education, multiple regression analysis with simultaneous entry was used, the results of which are mentioned below.

The results obtained from the table indicate that among the eight dimensions of the quality of life of educational coaches, the dimension of lack of physical performance with a beta coefficient of 0.807 is in the first degree of importance in terms of predicting the quality of clinical education and this value is at the alpha level. (0.01) was significant. Thus, the component of physical performance has been able to significantly predict the quality of clinical training of midwifery students. It can also be said that all the components of the variable of the quality of life of educational coaches have been able to explain (30.5) percent of the variable of the quality of life of educational coaches. Other dimensions did not have a significant contribution in predicting the quality of life of educational instructors. In other words, in predicting the quality of life of coaches, the contribution of other dimensions has been very insignificant (Table 6).

**Table 6:** Multiple regression to investigate the predicting power of quality-of-life dimensions of educators for the quality of education

Model	*β*	Standard *β*	error	*R*	*R* ^2^	t	Sig
Fixed	91.96		10.48	0.305	0.552	8.770	0.000
Physical performance	2.003	0.807	0.432			4.638	0.000
emotional health	0.108	0.023	1.067			0.101	0.920
the pain	1.835	0.049	4.967			0.369	0.713
general health	-1.289	-0.171	1.108			1.163	0.248
Energy and vitality	1,071	-0.160	1.568			0.683	0.497
Social Performance	0.090	-0.008	2.055			0.044	0.965
Limitations due to emotional problems	4.638	-0.153	1.366			1.032	0.305
Limitations due to physical health	1.160	-0.159	1.224			0.948	0.346

## Discussion

Among the most important components of clinical training, the characteristics of trainers during clinical training are considered as an important feature in the quality of training; The characteristics of educators can significantly influence the quality of education from the students’ perspective. In addition to professional competencies and interpersonal skills, the quality of life of instructors encompassing their physical, emotional, and social well-being may also affect their ability to deliver effective and engaging clinical training. Therefore, maintaining and improving educators’ quality of life can play a vital role in enhancing the overall quality of education. And the characteristics of professors can change the quality of education from the students’ point of view. In addition to these cases, the quality of life is another component that affects the physical and mental characteristics of coaches. Therefore, the level of quality of life may affect the quality of education [13]. Therefore, this study was conducted with the aim of determining the relationship between the quality of life of midwifery educators and the quality of clinical training of midwifery students in Fars’s province. The results of this study showed that based on the coefficients of the correlation test, there is no significant correlation between the quality of life of the coaches and their work experience and age. It is important to emphasize that the quality of clinical education is a complex and multifaceted construct influenced by numerous interacting factors, including institutional policies, curriculum structure, clinical environment, student motivation, prior academic preparation, and instructor pedagogical skills. Educators’ quality of life represents only one of these factors and may also interact with other variables as a mediator or moderator rather than acting independently. Therefore, the observed association in this study should be interpreted as relational rather than causal. Therefore, it can be concluded that educators’ quality of life is associated with the quality of clinical education at the institutional level, suggesting that educator well-being may play a contributory role in shaping students’ clinical learning experiences.

In line with these results, Rezaei et al. (2020) showed that the quality of life of professors is not affected by the professors’ age; However, in their study, they reported that professors with longer teaching experience had a higher quality of life, which is inconsistent with our findings [21]. In this regard, conflicting reports have been reported in studies. In Yang et al.‘s study, it has been shown that teachers who are between 30 and 40 years old have a lower quality of life than other age groups (30 and under and 40 and over) [22]. These differences can be due to differences in different statistical communities in the studies.

The results of this study showed that the dimension of physical performance has the greatest impact and the first degree of importance in the quality of life of midwifery professors in Fars’s province. In line with the results of this study, Yang’s study reported the physical dimension as a significant dimension of quality of life [22]. In the present study, the quality of life of the professors as well as the physical dimension is at a high level. In another study in Iran, Rezaei et al. reported that the level of the physical dimension is at a low level and the quality of life of professors is reported to be average [21]. Therefore, it can be said that the higher the physical health of the professors, the more likely it will have positive effects on their quality of life. The more personal interactions social support and security in the environment, the more quality of life and energy faculty members have [29]. On the other hand, studies have shown that self-care, which seems to be important for physical health, plays an important role in preventing physical diseases. Teachers with good self-care had better physical quality, which may be due to their high awareness of self-care [22]. It has also been reported in a different study that all aspects of quality of life are equally important and no significant difference was observed in terms of importance among them [30].

In clinical training courses, students learn and improve clinical skills in real conditions to prepare themselves for activity in the clinical environment [31]. In accordance with the next goal of the research, the results of this study showed that the quality of the student’s evaluation score was 96.98 out of 165 points of the general variable of the quality of clinical education, which is Table 2 the average and desirable level, which is in line with the studies conducted in science universities [20], and the victims and colleagues are based on the views of anesthesiology and operating room students, and the educational quality score is reported to be favorable. In the study of Rezaei et al. at the Islamic Azad University, the quality of clinical education was stated to be average [32]. The reason for this difference can be the educational environment and conditions of Azad University of Medical Sciences.

The results of this assessment showed that students assigned the highest scores to the dimensions of trainer performance and monitoring and evaluation, respectively. The educational environment received the lowest score in this assessment. The greatest importance was attributed to the instructor’s role, interactions with students, educational goals, and program design [33]. The most important areas were related to the teachers, the environment and the evaluator, respectively. Students and how to interact with Students in clinical environments, goals, and educational programs were reported. In these studies, it has been reported that the most important factor is the trainer and professors, which is in line with the results of the present study. Also, in other studies, the role of the instructor and professor has been reported as the highest factor in the quality of clinical education [34].

Goals and educational programs were introduced as the first factor, it is not consistent [35]. Also, in Sadeghi, et al.‘s study, among the average grades obtained by students in different areas of clinical education quality, the area of dealing with students was the most important factor affecting the quality of clinical education, and the area of evaluation with the lowest score was the least important factor from the student’s point of view [36], which was inconsistent with the results of the present study. This difference in dimensions can be due to the difference in conditions and facilities as well as different societies in different universities.

The results of this study showed that there is an inverse and significant correlation between the variable of clinical education quality of midwifery students and their age level. In other words, as the educational quality level of midwifery students increases, the age level of students decreases and vice versa. Therefore, younger students have a higher quality of clinical education than their older peers. In various studies, inconsistent results have been expressed regarding the demographic variables of students with the quality of clinical education. In another study, there was no relationship between gender, age, place of residence, and fields of clinical training [37]. No significant difference was observed between the variables of age, place of residence, academic year, and clinical training status [38].

In general, the results of this research with regard to the main goal of “determining the relationship between the quality of life of midwifery educators and the quality of clinical education from the point of view of midwifery students in Fars’s province” showed that according to the correlation coefficient, between the variable of clinical education of midwifery students and There is a positive and significant correlation between the quality of life of their educational coaches. In other words, in parallel with the increase in the quality of life of educational instructors, the level of clinical education of students has also increased and vice versa. Therefore, it can be concluded that educators’ quality of life may play a supportive and contributory role in shaping students’ clinical learning experiences, alongside multiple educational, organizational, and individual factors so that in addition to teaching the necessary skills, they are considered effective models and indicators for students. A capable instructor can make learning clinical experiences enjoyable for students by increasing their motivation [39].

Also, research conducted showed that factors such as the quality of the instructor and the quality of information and structural equations are factors that are effective in education [40]. There was no study in this field that directly measured the relationship between these variables. The studies that have been conducted on the quality of life of professors confirm the importance of the quality of life of professors in their work, which is in line with the results of the present study. In study, which was conducted with the aim of determining the relationship between the quality of life and academic productivity of Mazandaran University faculty members; showed that professors who had a better quality of life had higher academic productivity [30].

In summary, the findings demonstrate a significant positive correlation between midwifery educators’ quality of life and students’ perceptions of clinical education quality. These results highlight the critical role of instructors’ well-being in shaping students’ clinical learning experiences. Supporting educators’ physical and psychological health can therefore enhance the overall quality of midwifery education.

### Offers

Based on the results of this research, the following research are suggested (Table 7).

**Table 7:** Recommendations for future research

No.	Recommendation	Purpose / Expected Contribution
1	Conduct similar research in different provinces of Iran or other countries.	To improve the generalizability of the descriptive findings and confirm the observed relationships in diverse settings.
2	Investigate additional factors that may influence the quality of clinical education.	To provide a more comprehensive understanding of the determinants of clinical education quality.
3	Employ qualitative research methods in future studies.	To obtain deeper insights into educators’ quality of life and students’ perceptions of clinical education.

### Implementation problems in carrying out the plan and method of solving problems

The non-cooperation of the researched units in completing the questionnaires, for this purpose, the goals of the project were explained in order to attract their participation as much as possible. Another important limitation of this study is its descriptive–analytical and cross-sectional design, which does not allow for causal inference or control of all potential confounding and mediating variables. Factors such as institutional resources, instructor workload, teaching experience, student learning styles, and clinical setting characteristics were not modeled simultaneously. Future studies using multivariate, longitudinal, or structural equation modeling approaches are recommended to better elucidate the pathways through which educators’ quality of life may influence educational outcomes an additional limitation of this study is the absence of individual-level matching between specific instructors and specific students. Although educators and students were linked at the institutional level, individual instructor–student dyads were not analyzed. Consequently, the observed associations should be interpreted as contextual relationships within educational settings rather than direct individual-level effects.

## Data Availability

All data obtained from this study are included in the text of article.
